# Prioritizing CD4 Count Monitoring in Response to ART in Resource-Constrained Settings: A Retrospective Application of Prediction-Based Classification

**DOI:** 10.1371/journal.pmed.1001207

**Published:** 2012-04-17

**Authors:** Livio Azzoni, Andrea S. Foulkes, Yan Liu, Xiaohong Li, Margaret Johnson, Collette Smith, Adeeba bte Kamarulzaman, Julio Montaner, Karam Mounzer, Michael Saag, Pedro Cahn, Carina Cesar, Alejandro Krolewiecki, Ian Sanne, Luis J. Montaner

**Affiliations:** 1 Wistar Institute, Philadelphia, Pennsylvania, United States of America; 2 University of Massachusetts, Amherst, Massachusetts, United States of America; 3 BG Medicine, Waltham, Massachusetts, United States of America; 4 Royal Free Hampstead NHS Trust, London, United Kingdom; 5 UCL Medical School, London, United Kingdom; 6 University of Malaya, Kuala Lumpur, Malaysia; 7 University of British Columbia, Vancouver, British Columbia, Canada; 8 Philadelphia FIGHT, Philadelphia, Pennsylvania, United States of America; 9 University of Alabama, Tuscaloosa, Alabama, United States of America; 10 Fundación Huésped, Buenos Aires, Argentina; 11 University of the Witwatersrand, Johannesburg, South Africa; Harvard School of Public Health, United States of America

## Abstract

**Background:**

Global programs of anti-HIV treatment depend on sustained laboratory capacity to assess treatment initiation thresholds and treatment response over time. Currently, there is no valid alternative to CD4 count testing for monitoring immunologic responses to treatment, but laboratory cost and capacity limit access to CD4 testing in resource-constrained settings. Thus, methods to prioritize patients for CD4 count testing could improve treatment monitoring by optimizing resource allocation.

**Methods and Findings:**

Using a prospective cohort of HIV-infected patients (*n* = 1,956) monitored upon antiretroviral therapy initiation in seven clinical sites with distinct geographical and socio-economic settings, we retrospectively apply a novel prediction-based classification (PBC) modeling method. The model uses repeatedly measured biomarkers (white blood cell count and lymphocyte percent) to predict CD4^+^ T cell outcome through first-stage modeling and subsequent classification based on clinically relevant thresholds (CD4^+^ T cell count of 200 or 350 cells/µl). The algorithm correctly classified 90% (cross-validation estimate = 91.5%, standard deviation [SD] = 4.5%) of CD4 count measurements <200 cells/µl in the first year of follow-up; if laboratory testing is applied only to patients predicted to be below the 200-cells/µl threshold, we estimate a potential savings of 54.3% (SD = 4.2%) in CD4 testing capacity. A capacity savings of 34% (SD = 3.9%) is predicted using a CD4 threshold of 350 cells/µl. Similar results were obtained over the 3 y of follow-up available (*n* = 619). Limitations include a need for future economic healthcare outcome analysis, a need for assessment of extensibility beyond the 3-y observation time, and the need to assign a false positive threshold.

**Conclusions:**

Our results support the use of PBC modeling as a triage point at the laboratory, lessening the need for laboratory-based CD4^+^ T cell count testing; implementation of this tool could help optimize the use of laboratory resources, directing CD4 testing towards higher-risk patients. However, further prospective studies and economic analyses are needed to demonstrate that the PBC model can be effectively applied in clinical settings.

*Please see later in the article for the Editors' Summary*

## Abstract 2

Luis Montaner and colleagues retrospectively apply a potential capacity-saving CD4 count prediction tool to a cohort of HIV patients on antiretroviral therapy.

## Editors' Summary

**Background:**

AIDS has killed nearly 30 million people since 1981, and about 34 million people (most of them living in low- and middle-income countries) are now infected with HIV, the virus that causes AIDS. HIV destroys immune system cells (including CD4 cells, a type of lymphocyte and one of the body's white blood cell types), leaving infected individuals susceptible to other infections. Early in the AIDS epidemic, most HIV-infected people died within ten years of infection. Then, in 1996, antiretroviral therapy (ART) became available, and for people living in affluent countries, HIV/AIDS became a chronic condition. However, ART was expensive, and for people living in developing countries, HIV/AIDS remained a fatal illness. In 2003, HIV was declared a global health emergency, and in 2006, the international community set itself the target of achieving universal access to ART by 2010. By the end of 2010, only 6.6 million of the estimated 15 million people in need of ART in developing countries were receiving ART.

**Why Was This Study Done?:**

One factor that has impeded progress towards universal ART coverage has been the limited availability of trained personnel and laboratory facilities in many developing countries. These resources are needed to determine when individuals should start ART—the World Health Organization currently recommends that people start ART when their CD4 count drops below 350 cells/µl—and to monitor treatment responses over time so that viral resistance to ART is quickly detected. Although a total lymphocyte count can be used as a surrogate measure to decide when to start treatment, repeated CD4 cell counts are the only way to monitor immunologic responses to treatment, a level of monitoring that is rarely sustainable in resource-constrained settings. A method that optimizes resource allocation by prioritizing who gets tested might be one way to improve treatment monitoring. In this study, the researchers applied a new tool for prioritizing laboratory-based CD4 cell count testing in resource-constrained settings to patient data that had been previously collected.

**What Did the Researchers Do and Find?:**

The researchers fitted a mixed-effects statistical model to repeated CD4 count measurements from HIV-infected individuals from seven sites around the world (including some resource-limited sites). They then used model-derived estimates to apply a mathematical tool for predicting—from a CD4 count taken at the start of treatment, and white blood cell counts and lymphocyte percentage measurements taken later—whether CD4 counts would be above 200 cells/µl (the original threshold recommended for ART initiation) and 350 cells/µl (the current recommended threshold) for up to three years after ART initiation. The tool correctly classified 91.5% of the CD4 cell counts that were below 200 cells/µl in the first year of ART. With this threshold, the potential savings in CD4 testing capacity was 54.3%. With a CD4 count threshold of 350 cells/µl, the potential savings in testing capacity was 34%. The results over a three-year follow-up were similar. When applied to six representative HIV-positive individuals, the tool correctly predicted all the CD4 counts above 200 cells/µl, although some individuals who had a predicted CD4 count of less than 200 cells/µl actually had a CD4 count above this threshold. Thus, none of these individuals would have been exposed to an undetected dangerous CD4 count, but the application of the tool would have saved 57% of the CD4 laboratory tests done during the first year of ART.

**What Do These Findings Mean?:**

These findings support the use of this new tool—the prediction-based classification (PBC) algorithm—for predicting a drop in CD4 count below a clinically meaningful threshold in HIV-infected individuals receiving ART. Further studies are now needed to demonstrate the feasibility, clinical effectiveness, and cost-effectiveness of this approach, to find out whether the tool can be used over extended periods of time, and to investigate whether the accuracy of its predictions can be improved by, for example, adding in periodic CD4 testing. Provided these studies confirm its early promise, the researchers suggest that the PBC algorithm could be used as a “triage” tool to direct available laboratory testing capacity to high-priority individuals (those likely to have a dangerously low CD4 count). By optimizing the use of limited laboratory resources in this and other ways, the PBC algorithm could therefore help to maintain and expand ART programs in low- and middle-income countries.

**Additional Information:**

Please access these web sites via the online version of this summary at http://dx.doi.org/10.1371/journal.pmed.1001207.

- Information is available from the US National Institute of Allergy and Infectious Diseases on HIV infection and AIDS
- NAM/aidsmap provides basic information about HIV/AIDS and summaries of recent research findings on HIV care and treatment
- Information is available from Avert, an international AIDS charity, on many aspects of HIV/AIDS, including information on HIV/AIDS treatment and care and on universal access to AIDS treatment (in English and Spanish)
- The World Health Organization provides information about universal access to AIDS treatment (in several languages)
- More information about universal access to HIV treatment, prevention, care, and support is available from UNAIDS
- Patient stories about living with HIV/AIDS are available through Avert and through the charity website Healthtalkonline

## Introduction

Successful maintenance and expansion of anti-HIV-1 therapy programs in resource-limited settings is determined by multiple factors, such as clinical thresholds to start antiretroviral therapy (ART), drug access, trained personnel, and laboratory infrastructure. World Health Organization (WHO) guidelines for HIV-1 therapy in adults recommend initiation of anti-HIV-1 therapy after CD4 count drops below 350 cells/µl, with a clear indication to treat irrespective of clinical state if the CD4 count is below 200 cells/µl [1]. While the ideal monitoring of response to ART is dual (virological monitoring with high-sensitivity PCR as the benchmark to assess viral suppression, and monitoring of ART-mediated immune reconstitution via assessment of change in CD4 count [2]–[4]), this level of monitoring is often unsustainable within national health programs in resource-constrained settings because of the cost and limitations of the healthcare system infrastructure [5]–[11]. Although development of viral resistance linked to ineffective monitoring remains a concern in resource-poor settings, monitoring of clinical response (i.e., initial weight gain) and immune reconstitution (i.e., rise in CD4 cell counts) has been broadly used as a primary tool to assess success of therapy: there is a direct relationship between a lack of clinical response or a lack of a rise in CD4 count and risk of developing or not recovering from opportunistic infections. Indeed, WHO guidelines for patient monitoring address the imbalance between increasing treatment access and limited monitoring capacity by promoting therapy success definitions such as frequency of patients with CD4 count >200 cells/µl at 6, 12, and 24 mo after starting ART [1].

Despite the advent of newer, more cost-effective point-of-care devices for CD4^+^ T cell count determination using peripheral or capillary blood, the cost of laboratory-based CD4 count determinations to determine disease progression, indication for therapy, and response to ART remains high in terms of both economic and human resources (i.e., the need for specialized instrumentation and trained laboratory staff). Thus, numerous attempts have been made to identify low-cost surrogate markers that are widely available even in resource-limited settings, with the intent of eliminating the need for such intense CD4 count testing within resource/capacity-limited national HIV therapy programs [12]. The WHO recommends the use of total lymphocyte count to monitor untreated chronic HIV infection as a surrogate for disease progression changes, recommending treatment for patients with TLC<1,200 cells/µl [1]. While useful in the context of when to start treatment, TLC and other surrogate markers have not been shown to be useful in monitoring therapy response and/or treatment failure [13]–[19]. To date, no strategy has been proposed to reduce the need for CD4 testing after ART.

Using prediction-based classification (PBC) [20], a recently described model-based approach that accommodates repeatedly measured quantitative biomarkers for outcome prediction, we have developed a prioritization strategy to monitor response to ART based on baseline CD4 count, prospective white blood cell count (WBCC), and lymphocyte percent (Lymph%) measurements. In contrast to previous attempts focused on providing a direct surrogate marker for CD4 count, our approach could be used to direct limited healthcare resources to high-priority patients classified below predetermined CD4 count thresholds of clinical significance.

## Methods

### Cohorts

Anonymized data (WBCC, Lymph%, and CD4 count) were obtained from a cohort of 3,357 HIV-1-infected, ART-naïve individuals at the following clinical sites: Royal Free Hampstead NHS Trust, London, UK (used to generate the prediction rule); University of Alabama at Birmingham, Birmingham, Alabama, US; Jonathan Lax Center, Philadelphia FIGHT, Philadelphia, Pennsylvania, US; University of Malaya, Kuala Lumpur, Malaysia; University of the Witwatersrand, Johannesburg, South Africa; Fundación Huésped, Buenos Aires, Argentina; and University of British Columbia, Vancouver, British Columbia, Canada, for a total of 32,974 cumulative observations. Individual contributions from each site are summarized in Table 1. Participants were repeatedly observed for up to 3 y after ART initiation, and all patients had at least one post-initiation-of-ART (baseline) assessment. There were no restrictions on initial CD4 count.

**Table 1:** Cohort description.

Cohort	Site							
	Buenos Aires	London	Kuala Lumpur	Philadelphia	Johannesburg	Birmingham	Vancouver	Total
Total	100 (542)	270 (2635)	35 (102)	72 (399)	1,351 (4,239)	66 (640)	1,463 (24,336)	3,357 (32,893)
Cohort 1	58 (217)	214 (1,375)	15 (45)	55 (223)	654 (2,058)	59 (292)	901 (5,985)	1,956 (10,195)
Cohort 2	15 (147)	49 (679)	0 (0)	5 (43)	0 (0)	32 (398)	518 (8,559)	619 (9,826)

Data are expressed as number of individuals (number of observations over time). Cohort 1 is composed of individuals with complete data—between one and six assessments (CD4^+^ T cell count, WBCC, and Lymph% measured at the same time) within each 6-mo interval—for 1 y of follow-up. Cohort 2 is composed of individuals with complete data for 3 y of follow-up.

Primary analysis is focused on a subset of individuals (*n* = 1,956; Cohort 1) with complete data, defined as having at least one assessment (CD4^+^ T cell count, WBCC, and Lymph% measured at the same time) and no more than six assessments in each 6-mo period of follow-up—for 1 y after initiation of ART. Additionally, we consider a subset of these individuals (*n* = 619; Cohort 2) with complete data for 3 y of follow-up to assess the longer-term feasibility of this strategy.

### Statistical Analysis

We applied the PBC algorithm, which in brief involved fitting a mixed-effects model to the repeated measures of CD4 counts and, in turn, using model-derived estimates to define a prediction rule for whether post-baseline (start of ART) observations would be above predefined thresholds of 200 and 350 CD4^+^ T cells/µl. Patients with values predicted to be below these thresholds would be prioritized for actual CD4 testing. Algorithm performance compared to an alternative generalized linear modeling approach is detailed in Foulkes et al. [20] and includes improvements in sensitivity, positive predictive value, and negative predictive value for the same false positive rate (FPR). Briefly, the primary advantages of PBC over alternative strategies are the following: (1) PBC draws strength from the full range of continuous outcomes (through application of a linear model) while offering clinically relevant measures (such as positive and negative predictive value) through subsequent classification; and (2) it incorporates simultaneously multiple, repeatedly measured biomarkers observed at unevenly spaced intervals.

Formally, the PBC algorithm with cross-validation (CV) is given as follows, with additional details and formal mathematical derivations provided in Foulkes et al. [20]. Re-substitution estimates were determined using the same algorithm detailed below, with the full cohort used in place of both the learning and test samples (i.e., removing steps 1 and 7).

### PBC Algorithm

#### Step 1

We first randomly selected a learning sample composed of approximately 90% of the individuals in the full cohort to derive the prediction rule. Sample data included baseline (defined as time of ART initiation) and repeated measurements of CD4 count, WBBC, and Lymph% up to 3 y after ART initiation. The remaining approximately 10% of individuals made up the test sample.

#### Step 2

Based on the learning sample selected in step 1, we fitted a mixed-effects change-point model to repeatedly measured CD4 counts, with fixed effects for allowing different slopes before and after 1 mo on ART, and random person-specific intercept and slope terms (for time). The two time slopes are intended to reflect the rapid rise in CD4 count during the first month after ART initiation, and then the more gradual increase in CD4 over the remaining observation time. Additional fixed-effects terms for baseline CD4 count and baseline and time-varying WBCC and Lymph% are included as predictor variables. Also included in the model are fixed interaction effects between baseline CD4 count and (1) time before and after the change point, (2) baseline and time-varying values of WBCC, and (3) baseline and time-varying values of Lymph%. All terms in the model have Wald test statistic *p*-values of less than 0.10 for the complete cohort analysis, and main effects are included when corresponding interaction effects are statistically significant. Notably, application of a less stringent level 0.10 test is appropriate at this stage given that the algorithm additionally includes implementation of a CV procedure. Model fitting is performed using the lme() function of the nlme package in R version 2.11.1.

#### Step 3

Based on the model-based estimates derived in step 2, we calculated predicted values of CD4 count for all post-baseline observations in the learning sample. We also determined the lower bounds of corresponding one-sided level-α prediction intervals for a range of α values. Details about the calculation of these prediction intervals, including derivation of the prediction variance, as well as a discussion of their interpretation as credible intervals are provided in Foulkes et al. [20]. For a given α, the lower bound is denoted 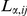 for the *j*th time point for individual *i*. A new binary predicted response, denoted 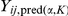 for the *j*th time point for individual *i*, is then defined as an indicator for whether the corresponding lower bound is greater than *K* where *K* = 200 or 350. That is, we let
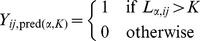
(1)for each CD4 threshold *K* = 200 and 350. We selected 200 CD4^+^ T cells/µl as indicative of a risk of development of opportunistic infections, and 350 CD4^+^ T cells/µl, the WHO-recommended ART initiation threshold [1], defining high-priority patients (i.e., patients requiring laboratory-based testing) as those failing to maintain CD4 counts above either of these thresholds after ART initiation.

#### Step 4

Again based on the learning sample, we compared the predicted variable 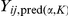 to an indicator for whether the observed CD4 count is greater than *K*, which we denote 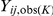, where
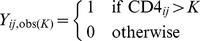
(2)and CD4*_ij_* is the observed CD4 count at the *j*th time point for individual *i*. To measure a prediction rule for a given α level and *K*, we calculated the FPR, defined as the proportion of post-baseline observations that fall below the threshold 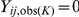 but are predicted to be above the threshold 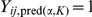 among those observed to be below the threshold.

#### Step 5

The “optimal” prediction rule was defined for a given threshold *K* as the rule (across all rules defined by the range of α values) that maximizes the FPR in the learning sample, subject to the constraint that the FPR is less than a predefined cut point. FPR cut points of 5% and 10% were considered clinically relevant. The α level corresponding to this optimal rule, denoted α^optimal^, was fixed for all subsequent analyses in the test sample.

#### Step 6

The test sample data were used to evaluate the optimal prediction rule as follows. Baseline CD4 counts, time since ART initiation, and baseline and post-baseline WBCC and Lymph% were used as inputs in the model derived in step 2 above to arrive at predicted CD4 counts at all measured post-baseline time points for individuals in the test sample. Notably, it is assumed that post-baseline CD4 count is not observed in the test sample, and so a correction for the empirical Bayes estimates from the mixed model is required, as described in Foulkes et al. [20]. Corresponding lower bounds for one-sided level-α^optimal^ prediction intervals were determined. Formally, this is denoted 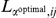 for the *j*th time point for individual *i* in the test sample. Binary predictions for all post-baseline CD4 counts within the test cohort were then defined according to Equation 1 where 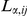 was replaced with 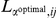. The dichotomized observed CD4 counts, as given by Equation 2, were compared to these binary predictions to arrive at the cross-validated estimates of sensitivity, specificity, positive predictive value, negative predictive value, and capacity savings. Capacity savings is defined as the ratio of tests spared by use of the model (i.e., the number across all individuals of post-baseline time points at which CD4 count is predicted to be above the *K* threshold, and thus a CD4 test would not be performed, divided by the total number of post-baseline time points).

Steps 1 to 6 are repeated ten times, and the average and standard deviation (SD) of the estimates listed in step 6 are reported as CV estimates. As these parameters are interdependent, CV estimates are not consistently lower (or higher) than re-substitution estimates using the full cohort. Copies of the R scripts used are available at http://people.umass.edu/foulkes/software.html.

## Results

### Cohort Description and Follow-Up

**Figure 1:**
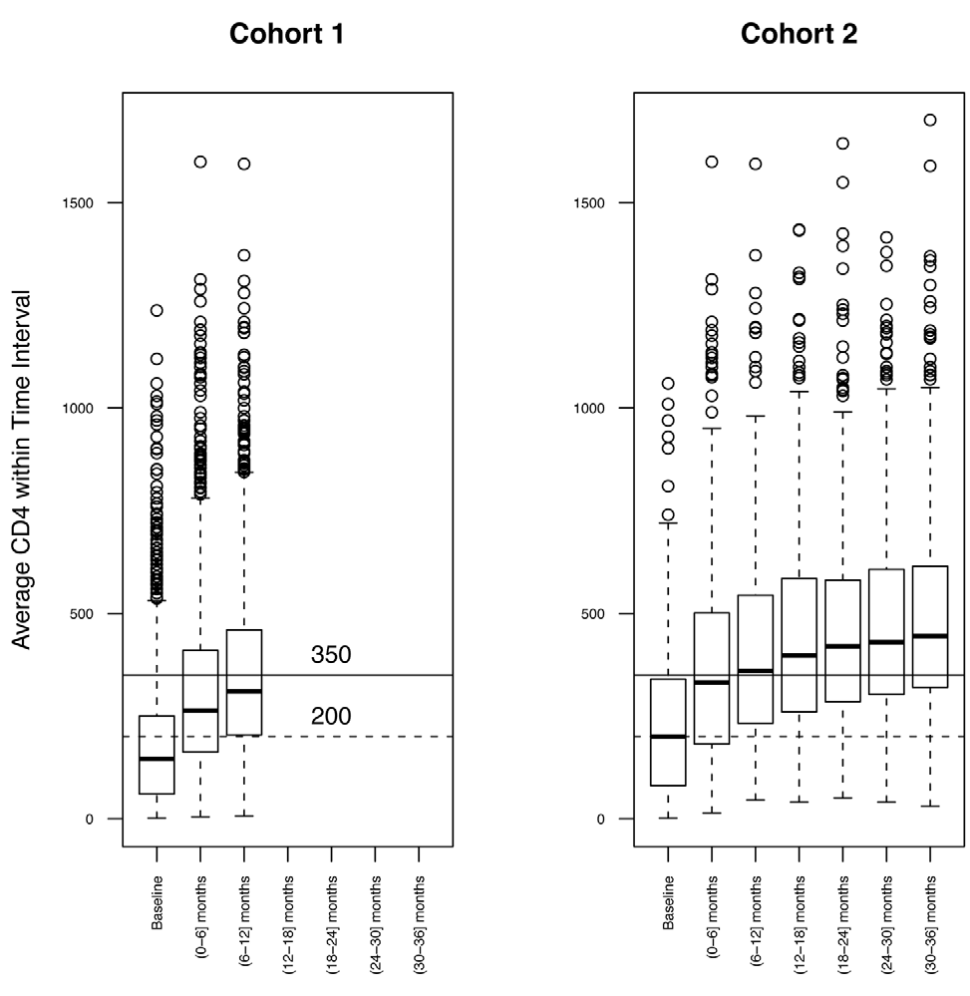
Distribution of CD4 count. The distribution of CD4 count at 6-mo time intervals was assessed for both Cohort 1 (left) and Cohort 2 (right). Means were calculated for patients with multiple CD4 count assessments in the same interval.

The geographical distributions of patients and corresponding numbers of observations over time are summarized in Table 1. For Cohort 1, the median baseline (pre-ART) CD4 count was 145.5 cells/µl; 34% of the patients initiated ART with a CD4 count >200 cells/µl, and 14.3% with a CD4 count >350 cells/µl. Median baseline WBCC was 4.7×10^3^ cells/µl, and median Lymph% was 30.7%. A detailed breakdown of these values for each cohort is summarized in Table 2. Unlike Lymph% and WBCC, median CD4 count and fraction of patients above 200 or 350 CD4^+^ T cells/µl were higher in the longer follow-up subset (200 for Cohort 2, as compared to 139 for Cohort 1), possibly due to the better clinical outcomes of patients that initiate treatment with higher CD4 counts, as well as the longer average follow-up in the London, Birmingham, and Vancouver cohorts. However, the rate of ART responders, defined as patients who had a documented CD4 raise of at least 20% from baseline over the follow-up time, was similar across all cohorts. The overall response to ART initiation was confirmed by the observed rise of median CD4^+^ T cell count over the observation time, as illustrated in 6-mo intervals in Figure 1 for the two cohorts.

**Table 2:** Baseline characteristics.

Cohort	CD4 Count (Cells/µl)					Lymph%		WBCC (Cells×10^3^/µl)	
	Median	IQR	Percent CD4 Counts>200 cells/µl	Percent CD4 Counts >350 cells/µl	ART Responders^a^	Median	IQR	Median	IQR
Total	139.0	49.0, 238.0	31.3	12.7		30.0	21.7, 38.5	4.7	3.6, 6.0
Cohort 1	145.5	60.0, 250.0	34.0	14.3	93.1	30.7	23.0, 38.9	4.7	3.7, 6.0
Cohort 2	200.0	80.5, 340.0	48.6	22.9	97.4	31.7	23.3, 40.0	4.6	3.7, 5.8

a Percent of patients with one or more post-baseline visits with CD4 count >1.2×baseline CD4.

IRQ, interquartile range (25^th^ percentile, 75^th^ percentile).

### PBC Application

The results of fitting mixed-effects change-point models (as described in step 2 of the PBC algorithm) to Cohorts 1 and 2 are given Table 3. The models suggest that the effects of WBCC, Lymph%, and time (before and after 1 mo on ART) are modified by baseline CD4 count (interaction terms are significant at the 0.10 level), and thus all main effects and interaction terms are included in the final model. The model-based estimates (coefficient estimates in Table 3) are used to derive the optimal prediction rule (as described in steps 3–5 of the PBC algorithm) using maximum FPRs of 5% and 10%. The results of applying the optimal rule to Cohort 1 data are given in Table 4. Of the 8,239 post-baseline observations, 5,976 (72.5%) had CD4 count >200 cells/µl, while 2,263 (27.4%) had CD4 count ≤200 cells/µl. Among observations with CD4 count ≤200 cells/µl, the algorithm correctly classified 2,037 (90%; CV estimate = 91.5%, SD = 4.1%); the corresponding FPR was 226/2,263 = 10% (CV estimate = 8.5%). Prioritized CD4 testing would be recommended for all observations with a predicted CD4 count <200 cells/µl (*n* = 3,729; 45.5%). The potential capacity savings based on this prioritization scheme, where the likelihood of not detecting a low CD4 count (FPR) is <10%, is 4,490/8,239 = 54.5% (CV estimate = 54.3%, SD = 4.2%). Alternatively, controlling the FPR at 5% would result in the option to prioritize testing for more observations (*n* = 4,705; 57.1%) and would result in a capacity savings of 42.9% (CV estimate = 44.4%, SD = 4.2%).

**Table 3:** Mixed-effects change-point modeling results for Cohort 1.

Cohort	Variable	Coefficient Estimate	Standard Error	*t*-Value	*p*-Value
**Cohort 1**	(Intercept)	−66.212	22.817	−2.902	0.004
	Baseline CD4 (BL_CD4)	1.011	0.101	10.053	0.000
	Time (in months)	37.424	19.331	1.936	0.053
	[Time−1]+^a^	−28.515	19.419	−1.468	0.142
	Baseline Lymph% (BL_Lymph%)	−0.119	0.318	−0.374	0.708
	Baseline WBCC (BL_WBCC)^b^	19.247	16.466	1.169	0.243
	Lymph%	2.012	0.212	9.484	0.000
	WBCC^b^	117.441	12.326	9.528	0.000
	BL_CD4*BL_Lymph%	−0.008	0.001	−5.662	0.000
	BL_CD4*BL_WBCC	−0.521	0.066	−7.899	0.000
	BL_CD4*Lymph%	0.007	0.001	8.635	0.000
	BL_CD4*WBCC	0.282	0.044	6.357	0.000
	BL_CD4*Time	0.139	0.079	1.745	0.081
	BL_CD4*[Time−1]+	−0.148	0.080	−1.850	0.064
**Cohort 2**	(Intercept)	−64.254	48.206	−1.333	0.183
	Baseline CD4 (BL_CD4)	0.890	0.221	4.031	0.000
	Time (in months)	63.075	39.389	1.601	0.109
	[Time−1]+^a^	−56.659	39.409	−1.438	0.151
	Baseline Lymph% (BL_Lymph%)	−0.730	0.672	−1.086	0.278
	Baseline WBCC (BL_WBCC)^b^	−46.320	47.386	−0.977	0.329
	Lymph%	2.245	0.253	8.865	0.000
	WBCC^b^	152.095	17.088	8.901	0.000
	BL_CD4*BL_Lymph%	−0.005	0.002	−2.111	0.035
	BL_CD4*BL_WBCC	−0.272	0.140	−1.950	0.052
	BL_CD4*Lymph%	0.003	0.001	3.832	0.000
	BL_CD4*WBCC	0.233	0.056	4.139	0.000
	BL_CD4*Time	0.320	0.189	1.691	0.091
	BL_CD4*[Time−1]+	−0.332	0.189	−1.756	0.079

a [Time−1]+ indicates the positive component of [Time−1], given as follow-up time after the first month for Time >1 mo, and 0 for Time ≤1 mo.

b WBCC is scaled by (divided by) a factor of ten.

**Table 4:** Observed and predicted values resulting from application of PCB to Cohort 1.

Predicted Value	Observed CD4 Count >200 cells/µl	Observed CD4 Count <200 cells/µl	Total
Predicted CD4 >200	4,264 (51.7%)	226 (2.7%)	4,490 (54.5%)
Predicted CD4<200^a^	1,712 (20.8%)	2,037 (24.7%)	3,749 (45.5%)
Total	5,976 (72.5%)	2,263 (27.5%)	8,239 (100%)

Data are expressed as number of observations (percent of total).

a Prioritized CD4 testing recommended for this group.

**Figure 2:**
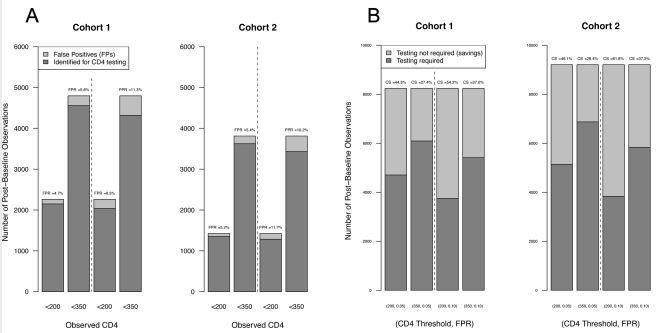
Summary of model performance. (A) Cross-validated estimates of FPRs. The bars represent the number of observed post-baseline observations below the thresholds indicated on the *x-*axis and at the indicated FPRs for Cohort 1 (left) and Cohort 2 (right). The dark shading indicates the number of observations correctly identified for laboratory-based CD4 testing (i.e., CD4 counts predicted to be and observed to be below threshold); lighter shading represents false positives (CD4 count incorrectly predicted as above threshold); cross-validated estimates of the FPRs are indicated above each bar. (B) Capacity savings (CS) estimates. Dark shading indicates the number of observations in Cohort 1 (left) and Cohort 2 (right) predicted to require laboratory-based CD4 testing (i.e., CD4 count predicted to be below threshold), and light shading the number of observations predicted to not require laboratory testing (i.e., CD4 count predicted to be above threshold) at the CD4 count threshold and FPR indicated below each bar.

These results, as well as the results from applying a 350-cells/µl threshold for CD4 count, are summarized in Figure 2 for both cohorts. Additional details on cross-validated parameter estimates, including sensitivity, specificity, negative predictive value, positive predictive value, and capacity savings, as well as corresponding SDs, are provided in Table 5 for both cohorts and thresholds. Extending this analysis to Cohort 2 (inclusive of 3 y of follow-up) resulted in overall similar capacity savings results, indicating that the model is applicable for at least 3 y from ART initiation without any intervening CD4 count assessment.

**Table 5:** Re-substitution and CV counts and estimates for the PBC model.

Cohort	*K* a	FPR^b^	Observed CD4 Count>*K*		Observed CD4 Count<*K*		Sensitivity^c^	Specificity^c^^,^ d	PPV^c^	NPV^c^	Capacity Savings^c^
			Predicted>*K*	Predicted<*K*	Predicted>*K*	Predicted<*K*					
1	200	0.10	4,264	1,712	226	2,037	0.71 (0.73; 0.048)	0.90 (0.92; 0.041)	0.95 (0.96; 0.020)	0.54 (0.57; 0.051)	0.54 (0.54; 0.042)
		0.05	3,421	2,555	113	2,150	0.57 (0.60; 0.050)	0.95 (0.95; 0.031)	0.97 (0.97; 0.018)	0.46 (0.49; 0.041)	0.43 (0.44; 0.042)
	350	0.10	2,348	1,094	478	4,319	0.68 (0.73; 0.044)	0.90 (0.89; 0.036)	0.83 (0.82; 0.048)	0.80 (0.82; 0.034)	0.34 (0.37; 0.039)
		0.05	1,908	1,534	239	4,558	0.55 (0.58; 0.064)	0.95 (0.94; 0.032)	0.89 (0.88; 0.057)	0.75 (0.76; 0.043)	0.26 (0.27; 0.039)
2	200	0.10	5,234	2,548	142	1,283	0.67 (0.71; 0.050)	0.9 (0.88; 0.087)	0.97 (0.97; 0.022)	0.33 (0.35; 0.053)	0.58 (0.62; 0.048)
		0.05	3,998	3,784	71	1,354	0.51 (0.53; 0.072)	0.95 (0.95; 0.043)	0.98 (0.98; 0.015)	0.26 (0.27; 0.041)	0.44 (0.46; 0.065)
	350	0.10	2,993	2,402	381	3,431	0.55 (0.55; 0.048)	0.90 (0.90; 0.034)	0.89 (0.89; 0.026)	0.59 (0.58; 0.048)	0.37 (0.37; 0.050)
		0.05	2,147	3,248	188	3,624	0.4 (0.41; 0.046)	0.95 (0.95; 0.027)	0.92 (0.92; 0.031)	0.53 (0.52; 0.049)	0.25 (0.26; 0.041)

a K: CD4^+^ T cell count threshold (cells/µl).

b FPR, assigned.

c Re-substitution estimate (mean CV estimate; SD of cross-validated estimates).

d Fixed, as determined by FPR.

NPV, negative predictive value; PPV, positive predictive value.

### Application Examples and Test Cost Comparison

**Figure 3:**
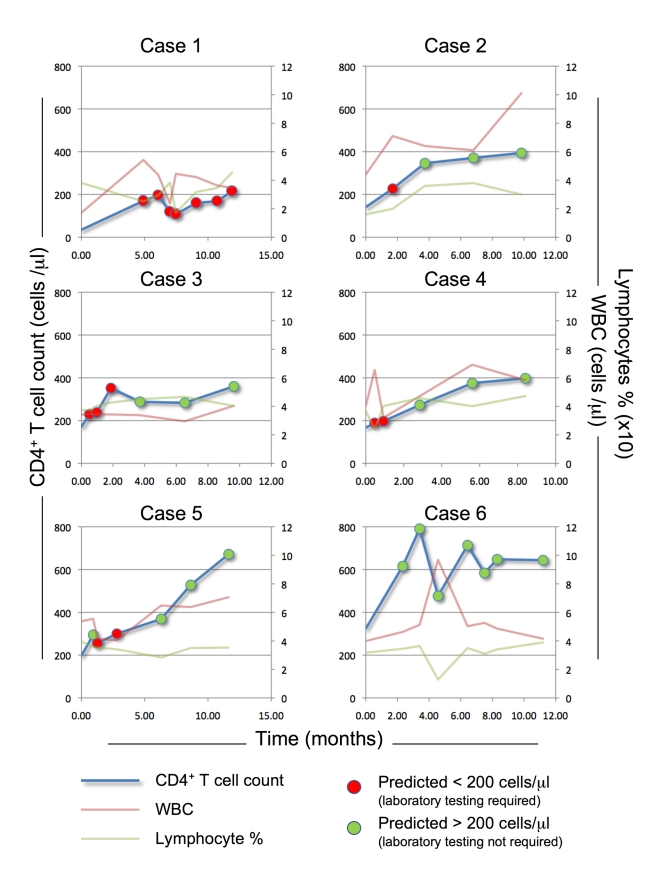
PBC predictive model application. The PBC predictive model (FPR 10%, CD4 threshold = 200 cells/µl) was applied to six patients form Cohort 2 (cases 1–6), selected to represent a range of baseline CD4^+^ T cell counts (low, case 1 and 2; medium, case 3–5; and high, case 6). The red and green lines represent assessed WBCC (WBC) and Lymph%, respectively; the blue line represents assessed CD4^+^ T cell count. The PBC algorithm application prediction at the corresponding visits is represented by red dots (predicted CD4^+^ T cell count ≤200 cells/µl, requiring laboratory-based testing) or green dots (predicted CD4^+^ T cell count >200 cells/µl, no laboratory-based testing required).

To illustrate the potential use of the model to provide individual predictions, we applied our algorithm to six representative individuals, selected from Cohort 2, based on initial CD4 count and the availability of multiple assessments. In the patients tested (Figure 3), the model performed well in prediction of a CD4 count >200 cells/µl (green dots); in fact, it was always correct in these cases, whereas, as expected, at some of the visits, patients predicted to have CD4 count ≤200 cells/µl (red dots) actually had a CD4 count >200 cells/µl (false negatives). As all cases predicted to be below threshold should be tested using the traditional laboratory-based methods, real time application of the model would not have exposed any of these patients to an undetected dangerous CD4 count, while sparing 20 (57%) of the 35 CD4 laboratory tests performed after baseline.

To assess the feasibility of the application of our modeling scheme to a healthcare setting scenario, we compared cost estimates for the PBC approach (at 200- and 350-cells/µl thresholds) to the estimated costs of a high-cost CD4 testing method (dual platform assessment) and a low-cost alternative (Guava platform, Millipore) as applied to a constrained-resource setting (South Asia). The details of this comparison are reported in Table S1. Briefly, according to our literature-based cost estimations, the PBC approach (considering a single initial laboratory-based CD4 assessment, followed by PBC application with a complete blood count performed for each individual, and CD4 assessment only for individuals predicted to have below-threshold CD4 values) could result in test cost savings when complete blood count cost is below “breakeven points” ranging from US$10.90 (PBC with 200-cells/µl threshold, CD4 laboratory testing based on dual platform assessment) to US$1.38 (PBC with 350-cells/µl threshold, Guava-platform-based laboratory CD4 testing). Importantly, the estimated cost of PBC (US$0.80) is well below all of these thresholds, suggesting that the PBC method may prove to be a cost-effective treatment monitoring strategy in future cost-effectiveness analyses.

## Discussion

We demonstrate that after obtaining a baseline WBCC and Lymph% and one laboratory-based CD4^+^ T cell assessment, the response to ART can be monitored relying on relatively low-cost clinical laboratory tests (i.e., WBCC and Lymph%) by using the PBC approach. This method enables us to predict whether CD4^+^ T cell count remains above predetermined safety thresholds with an estimated cross-validated FPR of 8.5%, and could potentially lead to testing capacity savings as compared to monitoring approaches based solely on repeated laboratory-based CD4 tests.

Given the high economic and capacity cost of laboratory-based CD4 assessments [21],[22], and the limited number of available accredited laboratories [23],[24]—circumstances that tax already stretched health systems as they implement national HIV treatment programs—a number of surrogate assessments (e.g., TLC) have been proposed to assess when HIV-infected patients require treatment, and to monitor them while they are undergoing treatment [12],[13],[15],[16],[25],[26]. Since to date these surrogates have not performed as well as CD4 counts in monitoring response to ART [13]–[19], two other options are open to improve current capacity utilization: (1) reducing the economic and human resource cost of performing laboratory-based CD4 tests, and/or (2) optimizing the use of existing resources to test only patients who are likely to need testing (i.e., patients likely to have dangerously low CD4 counts). The use of approaches that allow triaging patients at highest risk (e.g., patients who are failing treatment) for laboratory-based CD4 testing is expected to be particularly beneficial in resource-constrained settings characterized by high testing volume requirements (e.g., expanding treatment programs in sub-Saharan Africa).

Based on this premise, we conceived an algorithm that is intended as a triage/prioritization tool. To ensure the reasonableness of the approach, we fixed the acceptable FPR at 0.05 or 0.1. Although minimizing the FPR is desirable, there is an inevitable trade-off between the FPR and capacity savings. We believe that this error rate (5%–10%) is acceptable in light of the intrinsic variability of laboratory-based CD4 testing, and therefore clinically relevant.

Notably, erroneously predicting a CD4 count to be below 200 or 350 cells/µl when it is above this level (1−sensitivity) is less relevant to patient safety, as CD4 testing is recommended on predicted failures, thus eliminating the risk associated with this form of misclassification.

Our testing and validation indicate that the proposed model worked well over the 3-y follow-up time in our dataset. The possibility that periodic laboratory-based CD4 testing (e.g., every year) would improve and/or extend the predictive life of the model, to the point that it could be used continuously after ART initiation, remains open, as its determination will require dedicated prospective studies.

Our comparison of CD4 testing cost estimates indicates that the use of the PBC strategy is anticipated to result in a potential capacity and possibly cost savings at all the threshold levels assessed (Table S1). Further prospective healthcare economic studies modeling primary data (inclusive of all monitoring costs) obtained in target countries will be required to perform a net cost comparison, formally assessing the ramifications of the application and limitations of PBC testing for individual countries/regions' testing capacity, as well as long-term cost per outcome. While such studies are beyond the scope of this article, the data presented here provide a strong rationale for such studies.

Notably, the model-derived estimates and predictive rule are first derived based on application of PBC to a large cohort, as described in this article. Through development of publicly accessible web-based tools that incorporate the results presented herein, the above-described scenarios can be applied to single-case analyses. Additional contributions to this data resource will likely allow for further model refinement and improvements in predictive performance.

### Applications

In light of the considerations discussed above, our PBC-based tool could be useful in a number of scenarios.

#### Prioritization/triage of CD4^+^ T cell count testing at the laboratory level

In this case, a laboratory receiving a request for blood differential and CD4 count would first perform the differential, which requires limited time and commonly available resources; using the information obtained from this differential (WBCC and Lymph%), as well as the historic pre-ART baseline CD4 count (either stored or provided by the clinic), the laboratory could then run the prediction algorithm, and proceed to test only those patients who are predicted to have a CD4 count below a predetermined threshold.

#### Expansion of ART response monitoring at the clinic level

Due to cost limitations, some rollout programs allow only limited CD4 testing (e.g., every 6 mo). If ART-treated patients are additionally monitored at the rollout clinics for clinical visits and ART medication refills (e.g., every 3 mo), all patients could be monitored at these “non-CD4” visits for ART response by drawing a blood sample for a blood differential. Once the WBCC and Lymph% results are obtained from the laboratory, the clinic could employ the prediction tool to predict whether or not the patient's CD4 count is below a clinically meaningful threshold. Based on this, patients who are predicted to be failing treatment can be counseled for adherence, and/or further monitored by requesting a CD4 count. Because of the anticipated wider availability of complete blood count testing as compared to CD4 testing, this approach may result in shorter result turnaround time, partially reducing the acute need for point-of-care CD4 testing [27].

#### Reduction of confirmatory CD4 tests

Due to the intrinsic variability of current CD4 count tests, in many circumstances laboratory-based CD4 tests yield unexpected or doubtful results that are not in keeping with clinical observations (e.g., unexpectedly low CD4 count in a patient with increasing WBCC, lymphocytes, hemoglobin, or weight, and controlled viral load). In such cases, the CD4 count test may need to be repeated for confirmation before any clinically relevant action is taken (e.g., adherence counseling or regimen alteration and resistance testing). The use of the PBC method to independently confirm unexpected laboratory-tested CD4 counts could limit the need for repeated CD4 measures.

As indicated throughout this section, our conclusions should be tempered by considering some of the limitations of this work. First, this work is not intended to provide a complete analysis of the economic and healthcare outcomes of the application of PBC. Future prospective studies based in actual resource-constrained settings will be required to demonstrate the feasibility of this approach; here we focus on providing the foundation and rationale for such studies, which will be required to assess whether or not implementation of a PBC-based monitoring approach is a viable alternative to repeated CD4 testing. Second, it remains to be assessed how long this approach can be extended in time, e.g., by adding periodic CD4 testing, and whether or not periodic viral load testing would improve clinical outcomes of PBC-based monitoring. Third, PBC requires specifying an acceptable FPR. While the assessed rate can be lower, depending on the threshold used, the fact remains that the PBC has an intrinsic, small possibility of error. In light of the wide variability of CD4 count testing results, we do not consider this to be problematic, but it's possible that the collection of additional data (and possibly the introduction of other parameters, such as trends over time, into the model) might improve the model fit and improve its accuracy, and should be considered in future study design.

Finally, it is important to remark that the PBC-based method is not intended to substitute for laboratory-based CD4 testing, not to establish a “second tier” of healthcare standard to be applied to developing countries. Rather, we propose that this method is a potentially useful “triage” tool to direct available laboratory testing capacity to high-priority patients. As a tool to optimize the use of existing resources, the implementation of our PBC-based method would be most beneficial in settings where laboratory resources are currently limiting due to funding, human resources, or structural limitations.

### Conclusion

We propose a noninvasive, rapid turnaround method that could be applied to predict CD4 failure (i.e., a drop below clinically meaningful thresholds) in HIV-infected patients undergoing ART. By sparing up to 54% of current laboratory-based testing using a CD4 count threshold of 200 cells/µl, the implementation of our method could help focus laboratory-based CD4 count testing capacity on patients with higher likelihood of CD4 failure. This work provides the basis for future prospective testing of the model's overall safety, cost-effectiveness, and clinical outcomes in low-resource settings.

## Supporting Information

Table S1 **Comparison of monitoring cost estimates for PBC method versus high- and low-cost CD4 testing.**
(DOC)

## Abbreviations

ART: antiretroviral therapy
CV: cross-validation
FPR: false positive rate
Lymph%: lymphocyte percent
PBC: prediction-based classification
SD: standard deviation
TLC: total lymphocyte count
WBCC: white blood cell count: WHO, World Health Organization
