# Supplementary material for: Prioritizing CD4 Count Monitoring in Response to ART in Resource-Constrained Settings: A Retrospective Application of Prediction-Based Classification
Source: PLoS Med. 2012 Apr 17;9(4):e1001207. doi: 10.1371/journal.pmed.1001207 (PMC3328436; doi:10.1371/journal.pmed.1001207)
Supplement: Table S1 — Comparison of monitoring cost estimates for PBC method versus high- and low-cost CD4 testing. (DOC) [file pmed.1001207.s001.doc]

**Supplemental Table 1**: Comparison of cost estimates for PBC method vs. high and low-cost CD4 testing monitoring

| Laboratory test | Estimated cost (US$) ^1^ | Break-even CBC point | |
| --- | --- | --- | --- |
|  |  | PBC 200 | PBC 350 |
| Dual platform CD4 | 20 | 10.9 | 6.88 |
| Guava CD4 | 4 | 2.18 | 1.376 |
| Complete Blood Count ^2^ | 0.80 | n.a. | n.a. |

1. Cost estimates were obtained from the following publications:
   - *N. Kumarasamy et Al. J. Acquir. Immune Defic. Syndr. 2002 31:378-383*
   - *S. J. Goldie et Al. N. Eng. J. Med. 2006, 355:1141-1153*
   - *K. Pattanapanyasat et Al. Cytometry part B 2007, 72B:387-396*
   - *A. Kakar et Al. Indian J. Pathol. Microbiol. 2011, 54:107-111*
2. CBC inclusive of white blood cell count and lymphocyte %

Explanatory notes:

To compare the testing cost estimate for the PBC method against the cost of dual-platform and Guava-based CD4 testing, we determined the break-even (BE) cost point where PBC strategy cost = CD4 strategy cost. As the PBC strategy required CBC testing for all subjects to obtain a white blood cell count and lymphocyte %, and additional CD4 testing for subjects found below the “safety” threshold, PBC can be defined as:
 *CBC cost + (rate predicted below threshold x CD4 cost)*At break-even (B-E) point:
 *CBC cost = CD4 cost - (rate predicted below threshold x CD4 cost)*
According to this B-E analysis, the PBC strategy is anticipated to result in test cost saving when estimated CBC costs is below B-E point. In the case presented, based on available cost estimates from resource-constrained settings (India and Thailand, see below), the CBC estimated cost of US$ 0.80 was below B-E point for all strategies.

N.B.: this analysis is limited to the estimated cost of the laboratory tests. Further research and modeling in specific healthcare settings will be required to assess the impact of CD4 testing availability, capacity and other considerations such as costs related to false negatives.
